# Fully human IgG and IgM antibodies directed against the carcinoembryonic antigen (CEA) Gold 4 epitope and designed for radioimmunotherapy (RIT) of colorectal cancers

**DOI:** 10.1186/1471-2407-4-75

**Published:** 2004-10-15

**Authors:** Véronique Garambois, Fabienne Glaussel, Elodie Foulquier, Marc Ychou, Martine Pugnière, Robin X Luo, Binyam Bezabeh, André Pèlegrin

**Affiliations:** 1 INSERM, EMI0227 Centre de Recherche en Cancérologie, CRLC Val d'Aurelle-Paul Lamarque, F-34298 Cedex 5 Montpellier, France; 2 CNRS UMR 5160, Montpellier, France; 3 Abgenix, Inc., Fremont, CA, USA

## Abstract

**Background:**

Human monoclonal antibodies (MAbs) are needed for colon cancer radioimmunotherapy (RIT) to allow for repeated injections. Carcinoembryonic antigen (CEA) being the reference antigen for immunotargeting of these tumors, we developed human anti-CEA MAbs.

**Methods:**

XenoMouse^®^-G2 animals were immunized with CEA. Among all the antibodies produced, two of them, VG-IgG2κ and VG-IgM, were selected for characterization *in vitro *in comparison with the human-mouse chimeric anti-CEA MAb X4 using flow cytometry, surface plasmon resonance, and binding to radiolabeled soluble CEA and *in vivo *in human colon carcinoma LS174T bearing nude mice.

**Results:**

Flow cytometry analysis demonstrated binding of MAbs on CEA-expressing cells without any binding on NCA-expressing human granulocytes. In a competitive binding assay using five reference MAbs, directed against the five Gold CEA epitopes, VG-IgG2κ and VG-IgM were shown to be directed against the Gold 4 epitope. The affinities of purified VG-IgG2κ and VG-IgM were determined to be 0.19 ± 0.06 × 10^8 ^M^-1 ^and 1.30 ± 0.06 × 10^8 ^M^-1^, respectively, as compared with 0.61 ± 0.05 × 10^8 ^M^-1 ^for the reference MAb X4. In a soluble phase assay, the binding capacities of VG-IgG2κ and VG-IgM to soluble CEA were clearly lower than that of the control chimeric MAb X4. A human MAb concentration of about 10^-7 ^M was needed to precipitate approximatively 1 ng ^125^I-rhCEA as compared with 10^-9 ^M for MAb X4, suggesting a preferential binding of the human MAbs to solid phase CEA. *In vivo*, 24 h post-injection, ^125^I-VG-IgG2κ demonstrated a high tumor uptake (25.4 ± 7.3%ID/g), close to that of ^131^I-X4 (21.7 ± 7.2%ID/g). At 72 h post-injection, ^125^I-VG-IgG2κ was still concentrated in the tumor (28.4 ± 11.0%ID/g) whereas the tumor concentration of ^131^I-X4 was significantly reduced (12.5 ± 4.8%ID/g). At no time after injection was there any accumulation of the radiolabeled MAbs in normal tissues. A pertinent analysis of VG-IgM biodistribution was not possible in this mouse model in which IgM displays a very short half-life due to poly-Ig receptor expression in the liver.

**Conclusion:**

Our human anti-CEA IgG2κ is a promising candidate for radioimmunotherapy in intact form, as F(ab')_2 _fragments, or as a bispecific antibody.

## Background

During the last few years, radioimmunotherapy (RIT) using MAbs to specifically target therapeutic radiation doses to tumors has led to objective responses in radiosensitive hematological cancers, particularly, in non-Hodgkin's lymphoma (NHL) [1,2]. On the basis of these clinical results, ibritumomab tiuxetan (^90^Y-Zevalin; IDEC Pharmaceuticals) was registered for treatment of relapsed, indolent, and transformed CD20^+ ^NHL and, more recently, tositumomab (^131^I-Bexxar; Corixa) received regulatory approval; development of other promising products is in the pipeline [3].

Although targeting of solid tumors with radiolabeled antibodies was first reported years ago [4,5], RIT success in such tumors has been limited to patients with stable disease, occasional mixed responses, and serological responses [6-8]. Different parameters can be considered as responsible for these results: (i) the decreased radiosensitivity of solid tumors as compared with hematological cancers [9,10], (ii) the difficult penetration of MAbs in solid tumors [11], and (iii) consequently, the limited radiation dose that can be delivered to the tumor [12,13]. However, recent studies have reported a therapeutic window for RIT in solid tumors in small-volume and minimal residual disease [8] and in combination with chemotherapy [14]. The authors of all the recent pertinent clinical studies agree with the need of repeated injections for RIT of solid tumors and, consequently, with the need of humanized or, preferentially, human MAbs [14,15].

Colorectal cancers represent a high percentage of solid tumors and are dramatically in need of therapeutic progress. Surgery is the only potentially curative treatment. Despite recent developments in chemotherapy protocols, the overall median survival in metastatic colorectal cancer remains inferior to two years, and the recurrence rate after resection of a stage III tumor is up to 50% [16-18]. For RIT of colorectal cancers, carcinoembryonic antigen (CEA) is a preferential target antigen since (i) it is expressed in almost all tumors (>95%), (ii) it is available at high antigenic density on the cell surface, and (iii) many clinical studies have demonstrated a low MAb uptake in normal intestine despite CEA expression on these tissues. The only limitation of CEA as target antigen in RIT is the possible presence of circulating CEA in the serum of cancer patients, but this is without consequence in small-volume and minimal residual disease in which its level is generally low [19].

Different chimeric or humanized anti-CEA MAbs have been described and evaluated in experimental and clinical studies [8,14,15,19,20]. In the present study using the XenoMouse^® ^technology, we describe the generation and the characterization of two fully human anti-CEA antibodies, one IgG2κ and one IgM, designed for RIT of colorectal cancers.

## Methods

### Generation of fully human MAbs from XenoMouse^® ^strains

Generation and characterization of the XenoMouse^®^-G2 strain, engineered to produce fully Human IgG2κ antibodies, was described by Mendez et al. [21]. XenoMouse^®^-G2 animals were immunized i.p. with 20 μg of human recombinant CEA (rhCEA) [22] emulsified in complete Freund's adjuvant for the primary immunization and in incomplete Freund's adjuvant for additional immunizations carried out at one month intervals. Immunization was repeated three to five times. Two days before fusion, mice were boosted i.v. with 100 μg rhCEA in phosphate buffered saline (PBS). Spleen cells from immunized mice were fused with the non-secretory myeloma P3-X63-Ag.8.653 by addition of polyethylene glycol (PEG) and were subjected to HAT selection. Wells containing growing cells were evaluated for the production of the desired antibody, and if positive, the cultures were cloned. The hybridomas described in this report were subcloned at least five times.

### Reference anti-CEA MAbs and control MAb

The mouse-human chimeric MAb X4 was used as positive control in all the experiments. MAb X4 was constructed using the variable domains from the murine MAb CE25 and the constant domains from a human IgG_4κ _subclass [23,24]. It is specific for the CEA epitope Gold 4 [24] and does not cross-react with NCA or other granulocyte proteins [25]. Chimeric MAb X4 was produced in Sp2/0 cells transfected with a single vector containing both the chimeric heavy and light chains [24]. Murine MAbs F6, 35A7, B17, CE25, and 192, which are specific for the CEA epitopes Gold 1 to 5, respectively, were used for epitope determination [26]. MAb F6 was kindly provided in purified form by Schering-CIS Biointernational (Gif-sur-Yvette, France). MAbs 35A7, B17, CE25, and 192 were produced from mouse hybridoma ascites fluid by ammonium sulfate precipitation and ion-exchange chromatography. The human IgG MonoD, kindly provided by MAbgène (Alès, France), was used as irrelevant human IgG_1 _[27].

### Cell lines and human granulocytes

The CEA-positive human colon carcinoma LS174T cell line [28] was obtained from the Cell Distribution Center, American Type Culture Collection (Rockville, MD). The CO115-5F12 clone, obtained by transfection of the full-length CEA-cDNA in a CEA negative clone of the CO115 human colon carcinoma cell line, has been described [29]. Cells were grown in RPMI 1640 medium containing 10% heat-inactivated fetal bovine serum, streptomycin (0.1 mg/ml), penicillin (0.1 IU/ml), and amphotericin B (0.25 μg/ml). The neomycin analogue G418 was added at a concentration of 200 μg/ml to the CO115-5F12 cell culture. All culture medium supplements were purchased from Life Technologies, Inc. (Gibco BRL, Gaithersburg, MD). For flow cytometry analysis, cells were harvested after incubation for a few minutes in trypsin-EDTA (0.5 mg/ml and 0.2 mg/ml, respectively).

Granulocytes were obtained from heparin-treated human peripheral blood by using gradient density centrifugation methods. A double gradient was formed by layering an equal volume of HISTOPAQUE^®^-1077 and HISTOPAQUE^®^-1119. Following centrifugation at 700 g for 30 minutes, cells of the granulocytic series were found at the 1077/1119 interphase.

### Screening by ELISA and flow cytometry

The specificity of the antibodies in hybridoma supernatants was determined by ELISA using rhCEA to capture the antibodies (coating overnight at 2 μg/ml rhCEA at room temperature). Horse radish peroxidase (HRP)-conjugated goat anti-human IgG_κ _(Sigma, Lyon, France) and HRP-conjugated sheep anti-human IgG (γ chain) (Silenius, Hawthorn, Australia) were used as detection antibodies.

Determination of CEA or NCA specific antibodies in hybridoma supernatants was carried out by flow cytometry using CEA positive cells (CO115-5F12) and NCA positive cells (human granulocytes). About 5 × 10^5 ^cells were incubated for 1.5 h at 4°C with hybridoma supernatants or controls (RPMI medium for background measurement and RPMI containing 20 μg/ml MAb X4 for positive control). After washing, the cells were incubated with an FITC conjugated goat anti-human IgG kappa light chain (Sigma) or with the murine anti-human μ chain DA4-4 (ATCC HB-57), FITC labelled in our laboratory, for 1 h at 4°C; then they were washed twice before analysis on a FACScanII (Becton-Dickinson, Le-Pont-De-Claix, France). Each figure represents data obtained from analysis of 10000 cells.

### Human MAb production, purification, and molecular characterization

The percentage of fetal calf serum was gradually reduced in the culture medium before MAb purification (10, 5, 2.5 and 0%). MAbs were purified from large volumes of hybridoma supernatants or ascites produced in nude mice using Hitrap^® ^NHS-anti-human κ chain MAb HP6053 (ATCC CRLC-1758).

Proteins separated on SDS-PAGE 6% polyacrylmamide gels were transferred to a nitrocellulose membrane (Protran BA85, Schleicher and Schuell, Dassel, Germany). Non-specific binding sites were blocked overnight at 4°C by incubation with 5% (w/v) non-fat dry milk in TBS. The membrane was probed for 2 h at room temperature with serum diluted 1:1000 or the following antibodies: anti-human κ light chain-HRP conjugate (A7164, Sigma), anti-human λ light chain-HRP conjugate (A5175, Sigma), anti-human μ chain-HRP conjugate (A0420, Sigma), rabbit anti-human J chain-specific antiserum [30]. Bound serum antibodies were detected with a goat anti-rabbit whole molecule-alkaline phosphatase (AP) conjugate (A8025, Sigma). HRP was detected by addition of a chloronaphthol (Sigma) solution containing 0.05% of hydrogen peroxide and AP by addition of BCIP-NBT (Sigma). A human pentameric IgM anti-Rhesus D including a J chain, kindly provided by MAbgène (Alès, France), was used as positive control[27].

Antibody VH and Vκ cDNAs were recovered from hybridomas by RT-PCR and sequenced using the ABI-PRISM Big Dye Terminator Cycle Sequencing Kit (Perkin Elmer, Boston, MA). Determination of V, D, and J gene usage was performed using *in silico *methods.

### Measurement of antibody affinity to CEA

The affinities of the antibodies for CEA were determined by using surface plasmon resonance (SPR) technology (Biacore AB, Uppsala, Sweden). rhCEA [22] was immobilized on a CM5 sensor chip by the method of thiol ligation according to the manufacturer's instructions (BIACORE Methods Manual Supplement 5a). Each MAb was injected at a concentration of 50 μg/ml in HBS buffer (Hepes-buffered saline, pH 7.4, 3 mM EDTA ; 0.05% BIACORE surfactant) at a flow rate of 20 μl/min. Dissociation was carried out in running buffer (HBS). Regeneration of the sensor chip was performed by using 15 μl of 100 mM HCl. The kinetic parameters were determined by using BIAevaluation 3.2 software.

### MAbs and CEA radioiodination

Batches of 50 μg or 100 μg of MAb or rhCEA were labelled with 4.6 MBq or 11.5 MBq, respectively, of ^125^I or ^131^I, kindly provided by Schering-CIS Biointernational by the iodogen method (1,3,4,6-tetrachloro-3α, 6α-diphenylglycoluryl, Sigma). Free radioiodine was separated from the protein on a Sephadex G-25 column (Pharmacia) equilibrated in PBS, pH 7.4.

### Binding of MAbs to CEA in a soluble phase assay

About 1 ng ^125^I-rhCEA (final concentration 16.7 × 10^-12 ^M) was incubated with increasing concentrations of antibody (68.3 × 10^-12 ^to 33.3 × 10^-15 ^M) for 2 h in 0.15 M phosphate buffer, pH 7.4. CEA-antibody complexes were precipitated at 4°C with 53.5% (v/v) saturated ammonium sulfate. Background binding to an irrelevant human IgG, MonoD, was subtracted. The radioactivity precipitated with a rabbit polyclonal anti-CEA serum was taken as 100%.

### Epitope mapping by RIA

A competitive binding assay of radiolabeled human MAb and unlabeled anti-CEA MAb was used to determine the CEA epitope recognized by the human MAbs. RhCEA (100 ng/well) in TBS was coated on microtiter wells overnight at room temperature. An excess (500 ng/well) of each of the different reference anti-CEA MAbs, specific for the Gold epitopes 1 to 5, was then added to the wells and incubated for 1.5 h at 37°C. Then, without washing, each ^125^I-human MAb (15 ng) was added to the wells and incubated for 1 h 30 at 37°C. The percentage of binding was determined by measuring the radioactivity bound to the rhCEA after two washings.

### Biodistribution studies

Two million LS174T cells were grafted s.c. into the right flank of female Swiss nude mice (nu/nu, Iffa Credo, l'Arbresle, France). When the tumors had reached a volume of about 150 mm^3 ^(100 to 300 mm^3^), mice were grouped according to tumor volume. Lugol iodine solution (10% solution) was added to the drinking water one day before the injection of radiolabeled MAbs. Groups of four mice were injected with a ^125^I-labeled human MAb (VG-IgG2κ or VG-IgM) together with ^131^I-labeled chimeric MAb X4 as positive control. The total amount of each injected antibody was adjusted to 8 μg protein by adding unlabeled MAb. To determine the biodistribution of the MAbs, mice were sacrificed 24 or 72 h after injection. The blood, tumor, and all normal organs were weighed, and the differential radioactivity was measured in a dual channel scintillation counter. The results are expressed as the percentage of the injected dose of radioactivity present per gram of tissue (% ID/g).

## Results

### Human anti-CEA MAb characterization

In order to develop human anti-CEA MAbs, XenoMouse^®^-G2 animals were immunized with rhCEA. The XenoMouse^®^-G2 strain produces both fully human IgG2κ and fully human IgMκ antibodies as part of the normal immune response, as described by Mendez et al. [21]. Fusion of splenic B cells from immunized mice with mouse myeloma cells yielded a panel of hybridomas that secreted human anti-CEA antibodies as determined by ELISA and flow cytometry analysis (data not shown). These MAbs were produced for *in vivo *tumor targeting purposes. For this reason, (i) hybridoma supernatants were screened by flow cytometry because, by this technique, the selection is made on cell membrane-bound CEA, which is in a conformation as close as possible to that observed *in vivo*; and (ii) hybridoma supernatants were screened on human granulocytes to eliminate all hybridomas producing anti-NCA antibodies.

**Figure 1:**
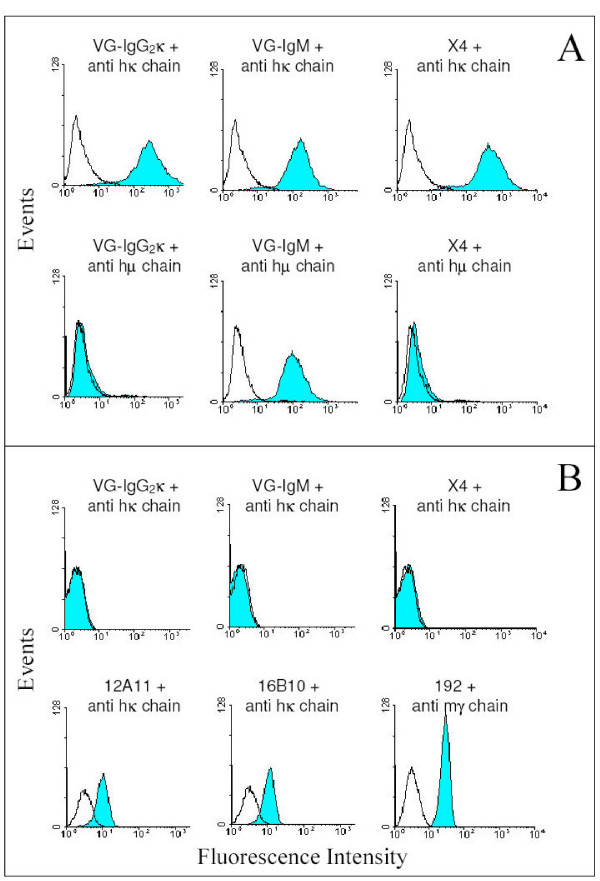
**Flow cytometry analysis. **Flow cytometry analysis of MAbs VG-IgG2κ, VG-IgM, and X4 reactivity against the CEA-expressing CO115-5F12 human colon carcinoma cell line (A) and NCA-expressing human granulocytes (B). 12A11 and 16B10 are two human anti-CEA MAbs that cross-react with NCA. 192 is a murine anti-CEA MAb that cross-reacts with NCA. Binding of the different primary antibodies was detected using either anti-human κ chain, anti-human μ chain, or anti-mouse γ chain as indicated.

Among the 52 antibodies produced, two, VG-IgG2κ and VG-IgM, were selected for further characterization based on hybridoma stability and flow cytometry analysis results. V_H _and V_L _domains sequencing confirmed the VG-IgG2κ and VG-IgM monoclonality and the isotype (data not shown). Flow cytometry analysis demonstrated strong binding of purified MAbs on CEA-expressing cells CO115-5F12 (Figure 1A) without any binding on NCA-expressing human granulocytes (Figure 1B).

MAb VG-IgG2κ and VG-IgM epitope specificities were determined in a competitive binding assay using five reference MAbs directed against the Gold 1 to 5 CEA epitopes [26]. As demonstrated by this assay (Table 1), the two human MAbs were directed against the CEA Gold 4 epitope since they were only inhibited by the murine MAb CE25 and the chimeric MAb X4. A partial inhibition, attributed to steric hindrance, was observed with MAb B17 (Gold 3) for VG-IgG2κ (31%) and X4 (13%).

**Figure 2:**
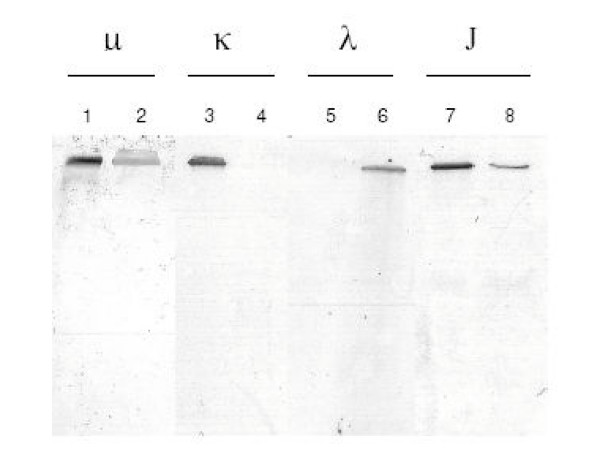
**Presence of J chain in VG-IgM. **Western blot analysis of VG-IgM (lanes 1, 3, 5, and 7) as compared with control IgM (lanes 2, 4, 6, and 8). MAbs transferred to a nitrocellulose membrane were analyzed for the presence of human μ heavy chain, human κ light chain, human λ light chain, and J chain as described in Materials and Methods.

VG-IgM was further analyzed for the presence of J chain using western blot. The J chain is a ligand for the poly-Ig receptor expressed on mouse hepatocytes. Figure 2 confirms that VG-IgM is constituted of human κ light chain, human μ heavy chain, and J chain as compared with an anti-Rhesus D IgM which also contains a J chain but is composed of a λ light chain [27].

### MAb binding to solid phase CEA and to soluble phase CEA

Using surface plasmon resonance technology, the affinities of purified VG-IgG2κ and VG-IgM were determined to be 0.19 ± 0.06 × 10^8 ^M^-1 ^and 1.30 ± 0.06 × 10^8 ^M^-1^, respectively, as compared with 0.61 ± 0.05 × 10^8 ^M^-1 ^for the reference MAb X4.

**Figure 3:**
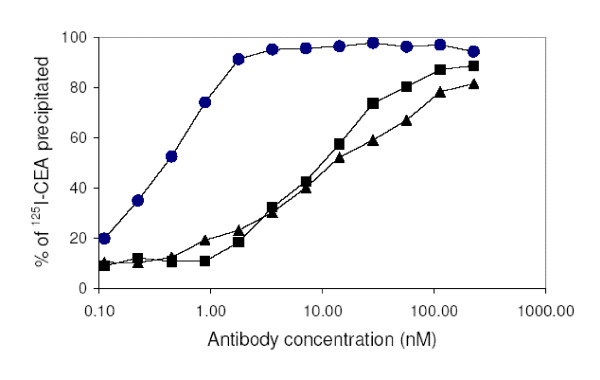
**Binding of MAbs to soluble phase CEA. **Precipitation of ^125^I-CEA with MAbs VG-IgG2κ (■), VG-IgM (▲), and X4 (●) following incubation in a soluble phase assay.

MAb binding to soluble phase CEA was measured using trace amounts of ^125^I-CEA incubated with increasing concentrations of antibodies (Figure 3). The binding capacities of VG-IgG2κ and VG-IgM to soluble CEA were found to be clearly lower than that of the control chimeric MAb X4. A human MAb concentration of about 10^-7 ^M was needed to precipitate around 1 ng ^125^I-rhCEA (final concentration 16.7 × 10^-12 ^M) as compared with 10^-9 ^M for MAb X4 (Figure 3).

### Biodistribution studies

**Figure 4:**
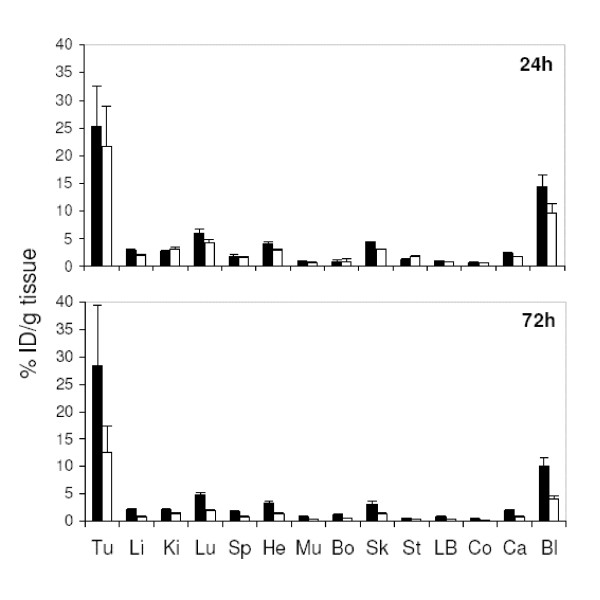
**Biodistribution studies in LS174T tumor bearing nude mice. **Biodistribution study of ^125^I-VG-IgG2κ (■) as compared with ^131^I-X4 (○) in LS174T tumor bearing nude mice dissected 24 and 72 h after i.v. co-injection. The tissues shown are (from left to right) tumor, liver, kidneys, lung, spleen, heart, muscle, bone, skin, stomach, low bowel, colon, carcass and blood. Results are expressed in terms of %ID/g ± SD.

The human MAbs were compared to the chimeric MAb X4 in nude mice bearing human colon carcinoma LS174T xenografts. Two groups of four mice were co-injected with ^125^I-VG-IgG2κ and ^131^I-X4. In the first group of mice dissected 24 h post-injection, ^125^I-VG-IgG2κ demonstrated a high tumor uptake (25.4 ± 7.3% ID/g), very close to that of ^131^I-X4 (21.7 ± 7.2% ID/g) (Figure 4). At 72 h post-injection, the ^125^I-VG-IgG2κ was still concentrated in the tumor with 28.4 ± 11.0% ID/g whereas the tumor concentration of ^131^I-X4 was significantly reduced, with only 12.5 ± 4.8% ID/g (Figure 4). This difference was attributed to a higher *in vivo *stability of VG-IgG2κ than X4 since the %ID recovered in the whole mouse at that time was 74.1 ± 1.5 and 55.6 ± 1.6 for the human and the chimeric MAbs, respectively. At no time after injection was there any accumulation of the radiolabeled MAbs in normal tissues. At 24 h, the tumor-to-normal tissue ratios of the antibodies were in the same range for ^131^I-X4, with representatives values of 10.98 ± 0.35, 28.78 ± 0.36, and 2.24 ± 0.38 for liver, muscle and blood, respectively, and for ^125^I-VG-IgG2κ, which gave values of 8.63 ± 0.30, 28.30 ± 0.31, and 1.74 ± 0.32 for the same organs, respectively (Table 2). At 72 h, the increase in the tumor-to-normal tissue ratios was very similar for the two MAbs (Table 2).

In two other groups of mice, ^125^I-VG-IgM was co-injected with ^131^I-X4. The reference chimeric MAb X4 gave tumor localization results comparable to that obtained when it was co-injected with ^125^I-VG-IgG2κ (19.1 ± 1.9 and 11.9 ± 3.1% ID/g tumor at 24 h and 72 h, respectively). Due to the mouse poly-Ig receptor expression in liver, ^125^I-VG-IgM was eliminated very rapidly, giving only 3.7 ± 1.0 and 0.3 ± 0.1% ID/g blood at 24 h and 72 h, respectively. This short clearance was responsible for a low tumor uptake (7.4 ± 2.8 and 1.8 ± 2.4% ID/g tumor at 24 h and 72 h, respectively). The percentage of injected MAb recovered in the whole mouse at 24 h was only 20.5% for ^125^I-VG-IgM as compared with 53.7% for ^131^I-X4.

## Discussion

Monoclonal antibodies are now routinely used in the clinic. Whereas the first generation of MAbs were murine or chimeric antibodies, it is now clear that the best clinical results are obtained with humanized or fully human MAbs [31]. Fully human antibodies such as ABX-EGF are anticipated to exhibit a long serum half-life and minimal immunogenicity with repeated administration, even in immunocompetent patients [32].

In some situations, naked MAbs have demonstrated their therapeutic efficacy, particularly, when the target antigen is a receptor implicated in cell proliferation processes [33,34]. However, the addition of radioactive isotopes on already efficient MAbs can lead to improved therapeutic results like that obtained with the anti-CD20 antibodies [35]. RIT remains attractive for solid tumors where the antibody penetration is limited and where the cross-fire phenomenon could lead to the destruction of cells which were not targeted by the radiolabeled MAb [13]. According to all the published or ongoing clinical studies, RIT could be applied to micrometastases from solid tumors or solid tumors in the minimal residual disease states [6,8,14,15]. One potential limitation of intact human MAbs for RIT could be their long serum half-life, which could lead to bone marrow suppression. The use of human MAb fragments for RIT would reduce their serum half-life, and possibly circumvent this limitation.

Among solid tumors, colorectal cancers represent one of the main causes of death, and CEA is well known as an ideal target antigen for RIT of these cancers [12]. Up to now, only a few human anti-CEA antibodies have been described. A human MAb directed against the carbohydrate moiety of CEA was described by Tsukazaki et al [36]. However, it was poorly characterized, and homology between the carbohydrate moieties of CEA and related molecules such as NCA would certainly lead to limited specificity of this anti-CEA MAb. Very recently, Imakiire et al. described human antibodies generated using the KM-Mouse [37]. They demonstrated complement- and cell-dependent cytotoxicity *in vitro *and presented preliminary data on tumor growth inhibition using MKN-45 cells grafted into SCID mice. However, they did not give any results on the biodistribution of their antibodies in radiolabeled form nor indications on how they could be used in RIT. Anti-CEA MAb PR1A3, which exhibits preferential binding to cell-bound CEA, was recently humanized, but to our knowledge, this MAb has not yet been evaluated in experimental or clinical studies [20]. A few clinical phase I or II trials suggest a certain degree of efficiency of humanized or chimeric anti-CEA mAbs, radiolabeled with either ^131^Iodine or ^90^Ytrium, in heavily pre-treated patients with metastatic colorectal cancer (MCRC) CT84.66 [8,14,15]. One of these trials showed a few objective tumor responses in MCRC of small-volume disease and provided some arguments in favor of this kind of therapy in an adjuvant setting [8]. The development of such chimeric, humanized, or human anti-CEA MAbs by different academic groups and industrial companies underlines the interest to generate a fully human MAb for RIT of colorectal cancers.

In the present study, the characterization of our fully human anti-CEA MAbs was conducted in comparison with the chimerized anti-CEA MAb X4, which has been shown to be clinically relevant [19,24]. Furthermore, the newly developed human MAbs were found to be directed against the same CEA epitope, namely Gold 4 (Table 1). That makes X4 an even better positive control, in particular, for the affinity measurements; although no difference has been reported between the different CEA epitopes for tumor immunotargeting [26]. VG-IgG2κ and VG-IgM are CEA-specific, i.e., NCA negative. This is particularly important for VG-IgM since the avidity generated by the pentameric molecule could have resulted in enhanced non-specific binding to CEA-related molecules.

The VG-IgG2κ and VG-IgM affinity constants were found to be similar to that of the control MAb X4 when determined using the BIACORE technology, but the binding of the human MAbs to soluble CEA (Figure 3) was clearly weaker than that of X4. This is particularly interesting for *in vivo *use in patients where some circulating CEA can be found. The molecular basis of this observation is not clear. The only possible comparison is with MAb PR1A3, which preferentially binds to cell bound CEA, and to a recombinant chimeric protein containing only the CEA B3 domain [20,38]. This reduced binding to the whole soluble CEA was attributed by the authors to be due to a conformational change supposed to occur when the CEA is shed into the circulation, resulting in steric blocking of antibody access to the B3 domain [20].

Using human colon carcinoma bearing nude mice, we obtained high tumor uptakes with VG-IgG2κ, making this antibody a good candidate for future clinical studies (Figure 4). In addition to the comparison with chimeric MAb X4, we also performed biodistribution studies comparing VG-IgG2κ and the murine MAb 35A7, with which we obtained the highest tumor uptakes in tumor bearing nude mice [39,40]. Seventy-two hours post injection, ^125^I-VG-IgG2κ localized in the tumor up to 26.2 ± 1.7% ID/g as compared with 28.5 ± 2.8% ID/g for ^131^I-35A7, suggesting a tumor residence time as long as that observed for MAb 35A7 (data not shown). These results could seem contradictory, given the fact that the affinity of VG-IgG2κ for CEA is not as high as that of Mab 35A7. This could be explained by the "affinity barrier" effect described in solid tumors by several authors who demonstrated that very high affinity MAbs localized at the periphery of tumor nodules and that lower affinity MAbs are able to distribute homogenously in these nodules [41-43].

Since, up to now, there are no data available on the biodistribution of any anti-CEA IgM in mice, we decided to analyze the biodistribution of our VG-IgM in LS174T tumor bearing nude mice. The disappointing tumor uptakes (7.4 ± 2.8 and 1.8 ± 2.4%ID/g tumor at 24 h and 72 h, respectively) could be attributed to a very short half-life due to poly-Ig receptor expression in the mouse liver which induces a rapid hepatobiliary transport of poly-IgA and IgM [44,45]. Based on the results obtained by Borchardt et al., we compared the tumor uptakes following i.p. and i.v. injection of the VG-IgM [46]. The tumor uptakes observed after i.p. injection were even lower (0.25%ID/g tumor at 24 h). These results are in contradiction with those obtained by Borchardt et al. [46]. In SK-Ov3 peritoneal carcinomatosis bearing nude mice, these authors showed a marked difference in tumor uptake between i.v. and i.p. injections of AC6C3-2B12 human IgM: 39% vs. 0.9%ID/g tumor for i.p. vs. i.v. injection, respectively, at 24 h and 28% vs. 1.4%ID/g at 48 h [46]. Liver and spleen uptakes were reduced following the i.p. injection as compared with the i.v. injection, but these uptakes in normal tissues could be related to this particular IgM and not relevant for our VG-IgM [46]. Indeed, the precise nature of the target antigen of their AC6C3-2B12 human IgM is unclear, but the discrepancy between our respective results could be due to the lack of J chain in their IgM. Without the J chain, IgM is unable to bind to the poly-Ig receptor, and as such it is not transported into the bile nor eliminated rapidly. The presence of the J chain in our VG-IgM makes it a fully functional IgM but limits its uptake by the human tumor grafted in nude mice.

## Conclusions

In the present study, we described two fully human anti-CEA MAbs. Even though the results obtained in tumor bearing nude mice cannot be extrapolated directly to humans, VG-IgM remains attractive for RIT of CEA-positive peritoneal carcinomatosis in man [47]since humans lack hepatic expression of poly-Ig receptor [48]. A first step toward this aim will be to study the biodistribution and tumor uptakes of low doses of radiolabeled VG-IgM. VG-IgG2κ is obviously a candidate for radioimmunotherapy in intact form, as F(ab')_2 _fragments, or as a bispecific antibody to be used in the affinity enhancement system (AES) approach [49]. Furthermore, we intend to test it in a model for immunophotodetection of cancer [50], and it could be the basis for preparing different anti-CEA immunoconjugates [51,52] and fusion proteins [53].

## Competing interests

No competing interest for VG, FG, EF, MY, MP and AP. RXL and BB are employees of Abgenix, Inc.

## Authors' contributions

VG participated in the design of the study, performed all the cell fusions, cell culture experiments, antibody characterization assays and *in vivo *experiments. FG and EF participated in the cell culture experiments and antibody characterization assays. MP performed the affinity measurements using BIACORE. MY participated in the design of the study. RXL and BB sequenced the V_H _and V_L _domains. AP conceived the study, participated in its design and coordination. All authors read and approved the final manuscript.

## Pre-publication history

The pre-publication history for this paper can be accessed here:



## Figures and Tables

**Table 1:** VG-IgG2κ and VG-IgM epitope mapping

		Tracer MAb		
		
Gold epitope	Inhibitor Mab	VG-IgG2κ	VG-IgM	X4
1	F6	1^a^	2	<1
2	35A7	<1	9	3
3	B17	31	<1	13
4	CE25	99	95	83
4	X4	98	89	68
5	192	3	11	<1

^a^Results are expressed as a percentage of inhibition of the binding of radiolabeled tracer MAbs by an excess of the reference MAbs directed against the five Gold CEA epitopes.

**Table 2:** Tumor to non-tumor tissue ratios of antibody concentrations

	24 h		72 h	
		
Tissue	VG-IgG2κ	X4	VG-IgG2κ	X4
Liver	8.63 ± 0.30^a^	10.98 ± 0.35	13.41 ± 0.41	15.80 ± 0.41
Kidneys	9.00 ± 0.30	7.13 ± 0.37	14.14 ± 0.42	10.33 ± 0.43
Lungs	4.23 ± 0.32	5.13 ± 0.36	6.03 ± 0.40	6.74 ± 0.41
Spleen	13.32 ± 0.33	13.48 ± 0.37	16.54 ± 0.42	17.53 ± 0.42
Heart	6.00 ± 0.30	7.34 ± 0.34	8.80 ± 0.41	10.00 ± 0.43
Muscle	28.30 ± 0.31	28.78 ± 0.36	36.48 ± 0.44	36.18 ± 0.45
Bone	31.15 ± 0.64	24.50 ± 0.68	26.14 ± 0.43	23.95 ± 0.42
Skin	5.74 ± 0.29	7.01 ± 0.33	9.10 ± 0.42	10.05 ± 0.43
Stomach	19.13 ± 0.31	12.64 ± 0.38	59.76 ± 0.46	40.04 ± 0.49
Small bowel	23.73 ± 0.30	26.12 ± 0.34	37.84 ± 0.42	41.97 ± 0.43
Large bowel	33.92 ± 0.29	33.57 ± 0.35	67.68 ± 0.42	68.45 ± 0.45
Carcass	10.39 ± 0.30	12.39 ± 0.34	15.39 ± 0.40	17.22 ± 0.42
Blood	1.74 ± 0.32	2.24 ± 0.38	2.81 ± 0.41	3.15 ± 0.42

^a^Ratios were calculated by dividing the %ID/g of the tumor by that obtained in each individual organ.
